# Health service use and work related outcomes in older adults with functional and cognitive impairments during the COVID-19 pandemic

**DOI:** 10.1186/s12889-025-25129-2

**Published:** 2025-11-07

**Authors:** Priya A. Prasad, Colin C. Hubbard, Irena Cenzer, John Boscardin, Himali Weerahandi

**Affiliations:** 1 Department of Medicine, Division of Hospital Medicine, University of California, 521 Parnassus Ave, Box 0131, San Francisco, CA 94143 USA; 2 Department of Medicine, Division of Geriatrics, University of California, San Francisco, CA USA; 3 Department of Epidemiology and Biostatistics, University of California, San Francisco, CA USA

**Keywords:** Long Covid, Older adults, Functional limitations, Cognitive limitations, Health services use, Health and retirement study

## Abstract

**Background:**

The COVID-19 pandemic had a lasting global health impact, with many survivors facing Long Covid. Older adults, already vulnerable to disability and cognitive decline, may also experience long-term challenges after COVID-19 infection. This study explores whether a history of COVID-19 infection interacts with pre-existing impairments in older adults, focusing on its effects on health services and work-related outcomes.

**Methods:**

This longitudinal cohort study used data from the Health and Retirement Study (HRS), spanning 2018 to 2022. Participants ≥ 50 years old in 2018 with documented functional and cognitive status scores and self-reported presence or absence of COVID-19 infection were included. Functional status was assessed using the Functional Limitation score, and cognitive status using the Crimmins cognitive scale or Langa scale if the HRS respondents were represented by a proxy. Health services use and work outcomes were evaluated using the 2022 HRS survey. Multivariable logistic regression models examined the association between baseline functional and cognitive status and outcomes, controlling for COVID-19 history, 2018 functional or cognitive status, age, gender, marital status, number of chronic conditions, household size, graduation from high school, and self-report of COVID-19 vaccination.

**Results:**

The study included 8,621 respondents. Those with severe functional limitations in 2018 were more likely to report health services use in 2022, irrespective of COVID-19 history. COVID-19 history did not significantly interact with baseline functional or cognitive impairments when evaluating health services use, ability to work, or disability benefit access. While older adults with moderate or severe functional limitations were more likely to report hospitalizations and nursing home stays, these outcomes were not significantly different based on COVID-19 history.

**Conclusions:**

In this cohort of older adults, the relationship between baseline functional and cognitive impairment with health services use, ability to work, or disability benefit access did not significantly vary by self-reported COVID-19 infection history. While COVID-19 may have long-term impacts on older populations, our data suggest that infection history alone did not amplify the effects of pre-existing impairments in those who survived the pandemic. Further research using validated measures of persistent symptoms is needed to understand how Long Covid may manifest in older adults.

**Supplementary Information:**

The online version contains supplementary material available at 10.1186/s12889-025-25129-2.

## Introduction

The COVID-19 pandemic has left a lasting impact on global health, with a significant number of survivors experiencing persistent symptoms that impact their daily lives and work capacity [1]. These enduring symptoms, nowknown as Long Covid [2], present a considerable challenge, particularly for older adults who already face higher risks of disability and cognitive decline.

Older age and pre-existing disabilities are well-documented risk factors for severe COVID-19 [3, 4], which in turn is associated with prolonged, debilitating symptoms [5]. Prior studies have shown that persistent symptoms after COVID-19 infection may lead to increased health services use [6, 7] and work impairments [8, 9] months after infection. However, these studies have largely focused on younger populations or individuals without baseline impairments, often lacking comprehensive data on pre-existing functional or cognitive limitations. As a result, it is unclear how long-term ramifications of COVID-19 infection may manifest in older adults with pre-existing impairments [2, 10], or whether these outcomes differ in meaningful ways based on COVID-19 history. Addressing this knowledge gap is needed to tailor healthcare, workforce, and social service interventions to meet the needs of this vulnerable population.

This study aims to bridge this gap by utilizing data from the Health and Retirement Study (HRS), covering the period from 2018 to 2022, to examine whether longitudinal health services use and work-related outcomes differ between older adults with and without a history of COVID and whether these outcomes are modified by baseline impairments. We hypothesize that older adults with pre-existing impairments who have a history of COVID-19 infection will exhibit increased healthcare utilization and reduced work capacity compared to those without a history of infection. By analyzing a unique dataset that includes comprehensive baseline assessments of functional and cognitive status, this research provides insights into the evolving needs for services among older adults after the COVID-19 pandemic.

## Methods

### Study population

This longitudinal cohort study utilized prospectively collected biannual data from the Health and Retirement Study (HRS) spanning from 2018 to 2022. The HRS is a longitudinal survey of adults aged 50 years or older and is sponsored by the National Institute on Aging. It is designed to create a representative cohort of community-dwelling older US adults [11]. The total response rate for the 2018 HRS wave was 74.4% and for the 2020 wave was 73.9%. Response rates for 2022 are not yet reported by HRS [12].

For our study, we included participants who were 50 years or older in 2018, had documented functional and cognitive status scores from the 2018 HRS survey, and responded to the 2018, 2020, and 2022 HRS surveys. Additionally, participants were required to have responded to a survey question on whether they had a COVID-19 infection from either the 2020 HRS survey or the 2021 Spring/Fall Perspectives on the Pandemic HRS survey [13]. The HRS 2021 Perspectives on the Pandemic survey was an additional mail-in survey administered to a subsample of over 20,000 respondents. This survey, fielded in two phases (spring and fall), was designed to assess how older adults were affected by the pandemic [13].

### Impact of COVID-19 on HRS

During the COVID-19 pandemic, data collection protocols for the HRS were modified. In particular, enhanced face-to-face (EFTF) interviews scheduled for the 2020 core wave were cancelled. Respondents who would have participated in EFTF interviews instead completed surveys by telephone or web [13].

### Measures

#### Functional and cognitive status

To determine functional status in 2018, we used the Functional Limitation score [14], based on the work of Yu and Zhang. Respondents’ answers across 12 questions on difficulty in performing the following tasks were summed: walking several blocks, jogging one mile, walking one block, sitting for about two hours, getting up from a chair after sitting for long periods, climbing several flights of stairs without resting, climbing one flight of stairs without resting, lifting or carrying weights over 10 pounds, stooping, kneeling or crouching, reaching arms above shoulder level, pushing or pulling large objects, and picking up a dime from the table. Items where the respondent had no difficulty were assigned a score of 0, and items where the respondent had some difficulty were assigned a score of 1. Total scores ranged from 0–12. Based on the distribution of scores from the 2018 HRS CORE interview respondents, the following categories were defined: those with less limitations (0–2), moderate limitations (3–7), and the most limitations (8–12).

To determine cognitive status in 2018, we used the Crimmins 27-point cognitive scale [15] which uses the immediate and delayed 10-noun free recall test, a serial 7 subtraction test, and a backward count from 20 test. Cut points for the Crimmins cognitive scale are defined as the following: normal (12–27), cognitive impairment—no dementia (7–11), and dementia (0–6). These categories are based on prior work from the Aging, Demographics, and Memory Study (ADAMS), an HRS sub-study of Alzheimer's disease and dementia that uses a 3-to 4-h in-home neuropsychological and clinical assessment as well as expert clinician adjudication to obtain a gold-standard diagnosis of cognitive impairment—no dementia or dementia [15].

For HRS respondents represented by a proxy, we used the 11-point scale described by Langa et al. [16] This scale uses the proxy’s assessment of the respondent’s memory ranging from excellent to poor (score, 0–4), the proxy’s assessment of whether the respondent had limitations in 5 instrumental activities of daily living (IADLs) (managing money, taking medication, preparing hot meals, using phones, and shopping for groceries; score, 0–5), and the survey interviewer’s assessment of whether the respondent had difficulty completing the interview because of a cognitive limitation (a score of 0–2 indicating, none, some, and prevents completion). Cut points here are defined as the following: normal (0–2), cognitive impairment—no dementia (3–5), and dementia (6–11). Again, these categories are based on prior work using the ADAMS dementia diagnosis as the gold standard, and correctly classifies 78% of HRS respondents as having dementia or not (76% of self-respondents and 84% of those represented by a proxy) [15].

#### Health services use and work outcomes

Health services use was evaluated with the 2022 HRS survey variables assessing whether the respondent had a nursing home stay, hospitalization, or saw a doctor in the last two years.

Work-related outcomes were evaluated by the 2022 HRS survey variables assessing whether the respondent was able to work, had a work-limiting impairment, or had applied for disability benefits from either the Supplemental Security Income (SSI) program and/or the Social Security Disability Insurance (SSDI) program. Patients who self-identified as “retired” during the 2018 HRS survey were excluded from the work outcomes analyses.

#### COVID-19 history

COVID-19 infection history was determined based on self-reported data from the 2020 HRS or the 2021 Fall/Spring Perspectives on the Pandemic HRS survey.

#### Sociodemographic and clinical variables

Age, sex, marital status, education level, race/ethnicity, smoking status, and self-reported chronic conditions (including diabetes, lung disease, emotional/psychiatric problems) were collected from the 2018 survey.

### Statistical analysis

#### Bivariate analysis

We first examined the bivariate relationships between COVID-19 history (negative vs. positive) stratified by functional or cognitive status and examined health services or work outcomes in 2022, using chi-square tests accounting for the survey design [17].

#### Multivariable analysis

Next, for health service use and work outcomes, we ran survey-weighted multivariable logistic regression models that incorporate inverse-probability weighting and design-based standard errors. Variables in the model included COVID-19 history, 2018 functional or cognitive status, age, gender, marital status, and number of chronic conditions. Separate models were constructed to evaluate the association between baseline functional and cognitive status and each outcome including health services use (doctor visits, hospitalizations, nursing home stays) and work outcomes (ability to work, work-limiting impairments, disability benefits applications). Interaction terms between COVID-19 history and baseline functional and cognitive status were tested to explore whether the impact of COVID-19 on outcomes varied by baseline impairment severity. Differences were considered significant for *p*-values < 0.05. All analyses were performed using R Statistical Software (v4.3.1; R Core Team 2023). Bivariate and multivariate analyses were performed using the survey (v4.4.1; Lumley 2004) and gtsummary (v2.1.0.9008; Sjoberg 2021) R packages [18, 19].

## Results

**Fig. 1:**
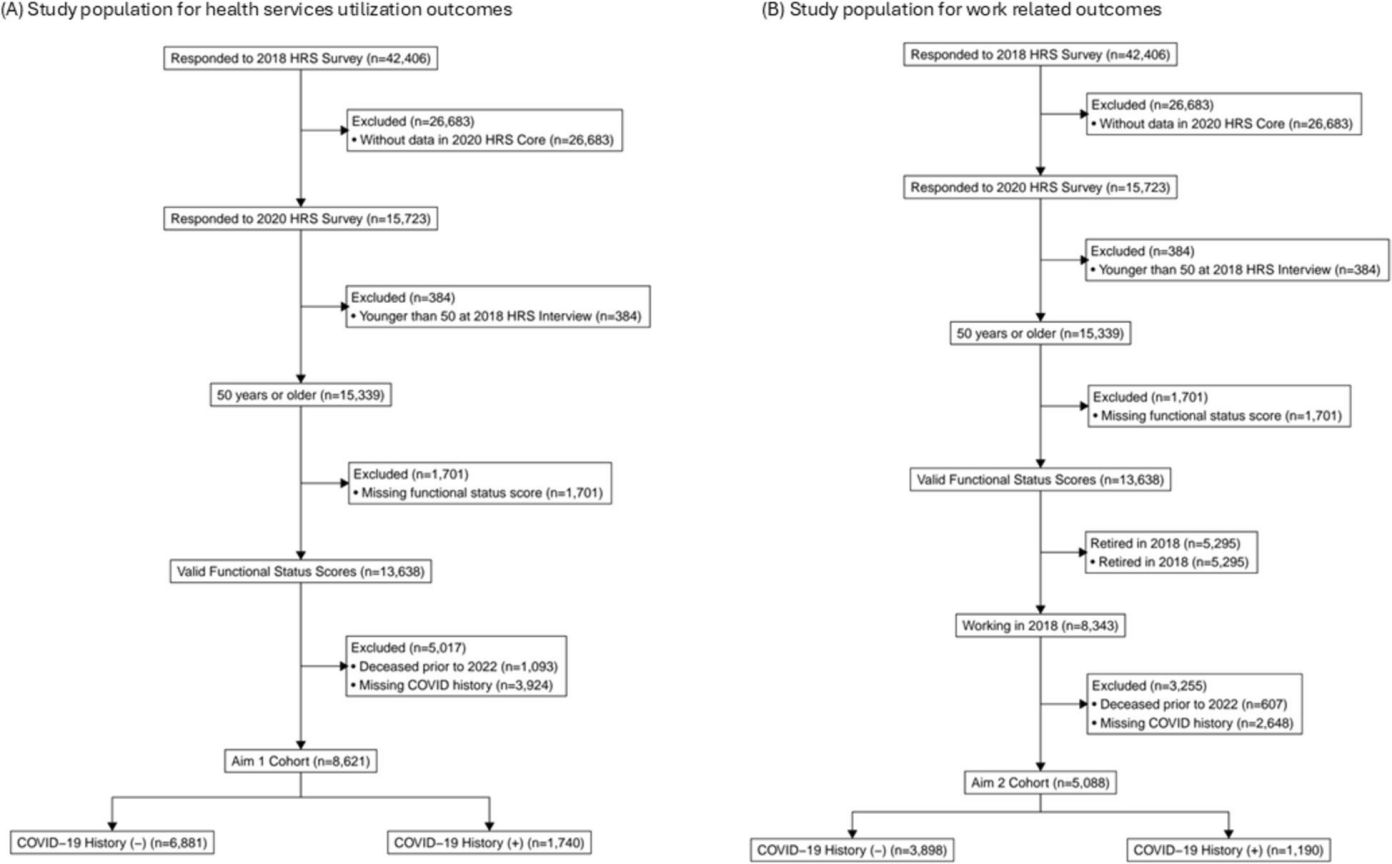
Study population flow diagram for the analyses of (**a**) healthcare utilization outcomes and (**b**) work related outcomes

For our study, we included participants who responded to the 2022, 2020, and 2018 surveys were 50 years or older and had valid functional and cognitive status scores from the 2018 HRS. Additionally, our cohort includes those who also responded to a survey question on whether they had a COVID-19 infection in either the 2020 HRS or the 2021 Fall/Spring Perspectives on the Pandemic HRS survey (*n* = 8,621). For work-related outcomes in this cohort, we included only individuals who reported working in 2018 (*n* = 5,088) (Fig. 1). We compared those who died or were lost to follow-up between the 2018 survey wave and the 2022 survey wave to those who were alive for the 2022 survey wave and found that those that died were less likely to have completed at least high school, less likely to be married, more likely to have cancer, lung disease, heart problems, and stroke, had a greater number of comorbidities, and were more likely to report being a current or former smoker (Supplemental Table 1).

### Baseline characteristics in 2018

Respondents with the most functional limitations tended to be older than the overall cohort, more likely to be women, have a comorbidity, or be a current smoker (Table 1). Black and Hispanic respondents were more likely to report the most functional limitations. On the other hand, respondents with less functional limitations were younger, more likely to have completed at least high school, more likely to be married, and more likely to be never smokers compared to the other functional limitation groups.

**Table 1:** Characteristics of cohort, stratified by 2018 functional limitation status

Characteristic	Functional Limitations				
	Overall *N* = 8621 *n* (%)^1^	Less *N* = 4566 *n* (%)^1^	Moderate *N* = 2804 *n* (%)^1^	Most *N* = 1251 *n* (%)^1^	*p*-value^2^
Age, years, median (IQR)	65 (59, 71)	63 (58, 69)	67 (60, 74)	67 (59, 74)	< 0.001
Female	5,161 (55%)	2,467 (50%)	1,779 (58%)	915 (68%)	< 0.001
Race					< 0.001
White/Caucasian	6,078 (83%)	3,318 (85%)	2,031 (84%)	729 (73%)	
Black/African American	1,690 (8.8%)	776 (7.1%)	520 (8.5%)	394 (18%)	
Other	825 (7.8%)	457 (8.0%)	243 (7.1%)	125 (9.0%)	
Hispanic	1,158 (7.7%)	577 (6.5%)	380 (8.6%)	201 (12%)	< 0.001
Completed at least high school	7,424 (91%)	4,118 (94%)	2,368 (89%)	938 (79%)	< 0.001
Married	5,579 (70%)	3,290 (76%)	1,701 (65%)	588 (50%)	< 0.001
Comorbidities					
Hypertension	5,204 (55%)	2,293 (45%)	1,908 (65%)	1,003 (76%)	< 0.001
Diabetes	2,272 (23%)	886 (16%)	881 (29%)	505 (37%)	< 0.001
Cancer	1,257 (14%)	555 (12%)	478 (16%)	224 (18%)	< 0.001
Lung disease	825 (8.6%)	180 (4.0%)	330 (12%)	315 (25%)	< 0.001
Heart problems	1,933 (21%)	707 (16%)	758 (26%)	468 (38%)	< 0.001
Stroke	574 (5.5%)	163 (2.6%)	218 (7.2%)	193 (16%)	< 0.001
Emotional/psychiatric problems	1,788 (21%)	565 (13%)	684 (26%)	539 (45%)	< 0.001
Arthritis	5,165 (58%)	1,905 (42%)	2,139 (76%)	1,121 (90%)	< 0.001
Total # of comorbidities, mean (SD)	2.05 (1.45)	1.51 (1.20)	2.57 (1.33)	3.45 (1.45)	< 0.001
Smoking					< 0.001
Never	4,112 (49%)	2,360 (52%)	1,236 (45%)	516 (39%)	
Former	3,555 (42%)	1,780 (40%)	1,268 (44%)	507 (44%)	
Current	924 (9.9%)	413 (7.9%)	293 (11%)	218 (17%)	

^1^All counts unweighted and percentages are weighted

^2^Design-based Kruskal–Wallis test for continuous variables; chi-squared test with Rao & Scott adjustment for categorical variables

Respondents with dementia were older than the overall cohort, less likely to be women, less likely to be married, and more likely to have a comorbidity, be current smokers, and have emotional/psychiatric problems (Table 2). Black and Hispanic respondents were more likely to report cognitive impairment-no dementia (CIND) and dementia in 2018.

**Table 2:** Characteristics of cohort, stratified by 2018 cognitive status

Characteristic	Cognitive Status				
	Overall *N* = 8621 *n* (%)^1^	Normal *N* = 7535 *n* (%)^1^	CIND *N* = 890 *n* (%)^1^	Dementia *N* = 196 *n* (%)^1^	*p*-value^2^
Age, years, median (IQR)	65 (59, 71)	64 (59, 71)	67 (60, 76)	68 (60, 77)	< 0.001
Female	5,161 (55%)	4,527 (55%)	522 (52%)	112 (47%)	0.10
Race					< 0.001
White/Caucasian	6,078 (83%)	5,523 (85%)	468 (67%)	87 (58%)	
Black/African American	1,690 (8.8%)	1,310 (7.5%)	301 (21%)	79 (28%)	
Other	825 (7.8%)	681 (7.4%)	115 (12%)	29 (14%)	
Hispanic	1,158 (7.7%)	901 (6.9%)	205 (17%)	52 (20%)	< 0.001
Completed at least high school	7,424 (91%)	6,763 (93%)	563 (71%)	98 (54%)	< 0.001
Married	5,579 (70%)	4,970 (71%)	510 (60%)	99 (50%)	< 0.001
Comorbidities					
Hypertension	5,204 (55%)	4,407 (53%)	647 (68%)	150 (78%)	< 0.001
Diabetes	2,272 (23%)	1,895 (22%)	302 (31%)	75 (36%)	< 0.001
Cancer	1,257 (14%)	1,098 (14%)	122 (13%)	37 (18%)	0.27
Lung disease	825 (8.6%)	659 (8.0%)	132 (14%)	34 (20%)	< 0.001
Heart problems	1,933 (21%)	1,633 (21%)	242 (28%)	58 (28%)	< 0.001
Stroke	574 (5.5%)	444 (4.8%)	92 (11%)	38 (25%)	< 0.001
Emotional/psychiatric problems	1,788 (21%)	1,485 (20%)	235 (27%)	68 (36%)	< 0.001
Arthritis	5,165 (58%)	4,444 (57%)	584 (64%)	137 (66%)	0.005
Total # of comorbidities, mean (SD)	2.05 (1.45)	2.00 (1.42)	2.56 (1.57)	3.08 (1.58)	< 0.001
Smoking					< 0.001
Never	4,112 (49%)	3,670 (49%)	366 (41%)	76 (34%)	
Former	3,555 (42%)	3,099 (41%)	373 (44%)	83 (48%)	
Current	924 (9.9%)	742 (9.4%)	147 (15%)	35 (18%)	

^1^All counts unweighted and percentages are weighted

^2^Design-based Kruskal–Wallis test for continuous variables; chi-squared test with Rao & Scott adjustment for categorical variables

### Functional/cognitive status in 2018 stratified by COVID-19 history in 2020/2021

There was a trend towards individuals with the most functional limitations in 2018 being more likely to report having COVID-19 in either 2020 or 2021 (13% v. 11%) compared to those with moderate or less functional limitations in 2018 (*p* = 0.056) (Table 3), although the difference was not statistically significant. Conversely, those with moderate functional limitations were less likely to report having COVID-19 in 2020 or 2021. For those with cognitive limitations in 2018, there was no significant difference in whether they got COVID in 2020 or 2021, compared to those with normal cognitive status.

**Table 3:** Functional/Cognitive status in 2018 stratified by COVID-19 infection history in 2020/2021

Characteristic	COVID-19 History in 2020/2021		
	Negative *N* = 6881 n (%)^1^	Positive *N* = 1740 n (%)^1^	*p*-value^2^
Functional limitations			0.056
Less	3,648 (58%)	918 (59%)	
Moderate	2,276 (31%)	528 (28%)	
Most	957 (11%)	294 (13%)	
Cognitive limitations			0.23
Normal	6,039 (92%)	1,496 (90%)	
CIND	694 (6.9%)	196 (7.6%)	
Dementia	148 (1.4%)	48 (1.9%)	

^1^All counts unweighted and percentages are weighted

^2^chi-squared test with Rao & Scott adjustment

### Functional impairment, COVID-19, and health services use

**Fig. 2:**
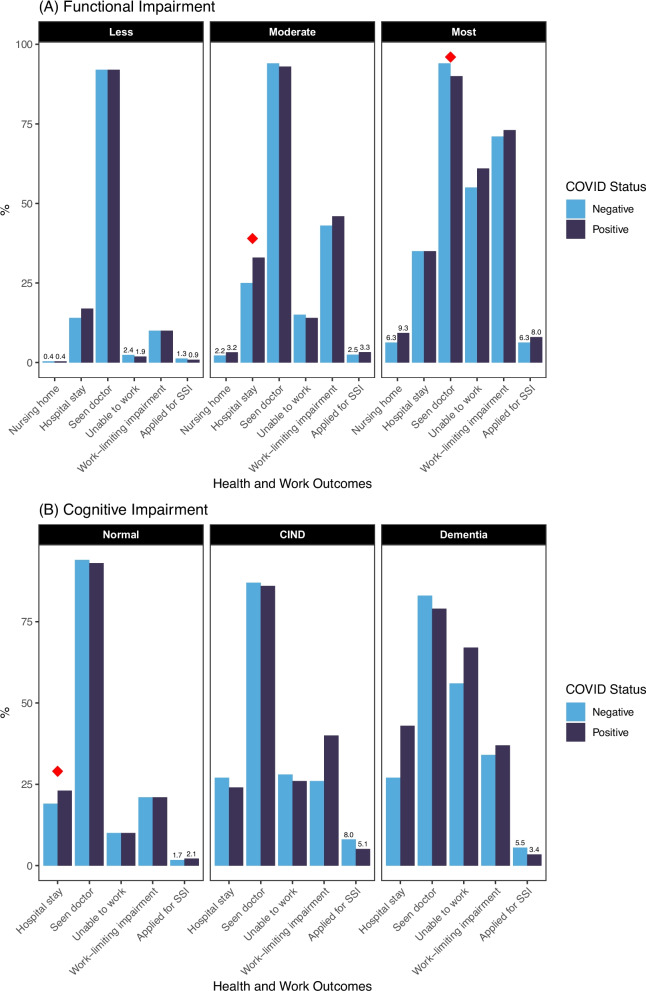
Health Services Usage and Work Ability in 2022 by COVID-19 Infection History, Stratified by (**a**) Functional Limitations in 2018 and (**b**) Cognitive Limitations in 2018

In bivariate analyses (Fig. 2), in those with less functional limitations in 2018, there was no difference in health services use in 2022 for those who reported a history of COVID-19 infection compared to those who did not have COVID-19.

In those who had moderate functional limitations in 2018, respondents who had COVID-19 were more likely to report in their 2022 survey that they had a hospitalization in the last 2 years compared to those who did not have COVID-19 (33% v. 25%, *p* = 0.006), but there was no difference in nursing home use or having seen a doctor.

In respondents with the most functional limitations, those who had COVID-19 were less likely to report having seen a doctor at least once in the last 2 years (90% v. 95%, *p* = 0.022), but there was no difference in either nursing home use or hospitalization.

In multivariable analyses, patients with moderate or the most functional limitations in 2018 were more likely to report a nursing home stay in the last 2 years (Table 4); however, that association was not modified by COVID-19 history (Moderate *p* = 0.640; Most *p* = 0.585, Supplemental Table 2). Similarly, patients with moderate or the most functional limitations were slightly more likely to report hospitalization in the last 2 years (Table 4), but again, there was no interaction with COVID-19 history (Moderate *p* = 0.429; Most *p* = 0.263, Supplemental Table 2). For a doctor visit in the past 2 years, there was no difference by functional limitation.

**Table 4:** Odds Ratios from multivariable analysis of health services usage and work ability in 2022 by COVID-19 infection history and functional limitation in 2018

	Ever stayed in nursing home	Ever stayed in the hospital	Seen a doctor	Unable to work	Work limiting impairment	Applied for SSI
COVID						
No	Ref	Ref	Ref	Ref	Ref	Ref
Yes	1.79 (1.10, 2.90)	1.66 (1.16, 1.58)	1.01 (0.76, 1.35)	0.85 (0.61, 1.17)	1.04 (0.79, 1.36)	0.88 (0.48, 1.61)
Functional Limitation in 2018						
Less	Ref	Ref	Ref	Ref	Ref	Ref
Moderate	3.18 (1.73, 5.86)	1.56 (1.33, 1.84)	1.15 (0.87, 1.52)	5.08 (3.11, 8.30)	5.02 (3.85, 6.55)	1.51 (0.74, 3.07)
Most	8.00 (4,10, 15.6)	2.00 (1.53, 2.61)	1.18 (0.74, 1.89)	32.8 (19.2, 56.2)	11.3 (7.27, 17.7)	2.70 (1.30, 5.62)

*Variables in the model included COVID-19 history, 2018 functional or cognitive status, age, gender, marital status, number of chronic conditions, household size, graduation from high school, and self-report of COVID-19 vaccination. SSI = supplemental security income

### Cognitive impairment, COVID-19, and health services use

In bivariate analyses (Fig. 2), those with normal cognitive function in 2018 who also had COVID-19 in 2020/2021 were more likely to report in their 2022 survey that they had a hospitalization in the last 2 years (23% v. 19%, *p* = 0.006). In patients with CIND, there was no significant difference in health services use reported in the 2022 survey in those with a history of COVID versus those without. Because few respondents with CIND or dementia were living outside of nursing homes in 2020, we did not examine nursing home usage.

In multivariable analyses, cognitive status was not associated with hospitalization (Table 5), but those with CIND and dementia were less likely to report having seen a doctor when compared to those with normal cognitive function (CIND: OR 0.59, 95% CI 0.42, 0.81); dementia: OR 0.46, 95% CI 0.22, 0.99). There was no interaction between cognitive impairment and COVID-19 history and health services use (Supplemental Table 2).

**Table 5:** Odds ratios from multivariable analysis of health services usage and work ability in 2022 by COVID-19 infection history and cognitive limitation in 2018

	Ever stayed in the hospital	Seen a doctor	Unable to work	Work limiting impairment	Applied for SSI
COVID					
No	Ref	Ref	Ref	Ref	Ref
Yes	1.36 (1.16, 1.59)	1.03 (0.77, 1.36)	0.87 (0.65, 1.15)	1.01 (0.79, 1.29)	0.90 (0.50, 1.62)
Cognitive Impairment in 2018					
Normal	Ref	Ref	Ref	Ref	Ref
CIND	1.15 (0.91, 1.46)	0.59 (0.42, 0.81)	2.34 (1.61, 3.38)	1.21 (0.75, 1.95)	2.85 (1.63, 4.97)
Dementia	1.17 (0.66, 2.07)	0.46 (0.22, 0.99)	10.1 (4.99, 20.5)	1.28 (0.44, 3.66)	1.18 (0.41, 3.38)

*Variables in the model included COVID-19 history, 2018 functional or cognitive status, age, gender, marital status, number of chronic conditions, household size, graduation from high school, and self-report of COVID-19 vaccination. SSI = supplemental security income; CIND = cognitive impairment-no dementia

### Functional impairment, COVID-19, and work related outcomes

In bivariable analyses (Fig. 2), there was no significant difference in reported ability to work, experiencing a work-limiting impairment, or applying for disability benefits in 2022 for those who reported a history of COVID-19 infection compared to those who did not have COVID-19 in any of the three categories of 2018 reported functional limitation (less, moderate, most).

In multivariable modeling (Table 4), those with increased functional limitations in 2018 were more likely to report being unable to work (less: referent; moderate: OR 5.08, 95% CI 3.11, 8.30; most: OR 32.8, 95% CI 19.2, 56.2) and more likely to have a work-limiting impairment (less: referent; moderate: OR 5.02, 95% CI 3.85, 6.55; most: OR 11.3, 95% CI 7.27, 17.7) after controlling for COVID-19 history, 2018 functional or cognitive status, age, gender, marital status, number of chronic conditions, household size, graduation from high school, and self-report of COVID-19 vaccination. Only those in the category of “most” functional limitation in 2018 were more likely to report applying for disability benefits in 2022 (most: OR 2.70, 95% CI 1.30, 5.62). However, there was no interaction between functional limitation and COVID-19 history and work outcomes (Supplemental Table 3).

### Cognitive impairment, COVID-19, and work related outcomes

In bivariable analyses (Fig. 2), when observing those who reported normal cognitive function in 2018, there was no significant difference in ability to work, experiencing a work-limiting impairment, or applying for disability benefits in 2022 when stratifying by COVID-19 infection in 2020/2021.

When observing those who reported having dementia in 2018, there was no significant difference in ability to work, experiencing a work-limiting impairment, or applying for disability benefits in 2022 when stratifying by COVID-19 infection in 2020/2021.

In multivariable modeling (Table 5), those with increased severity of cognitive function in 2018 were more likely to report being unable to work (no impairment: referent; CIND: OR 2.34, 95% CI 1.61, 3.38); dementia: OR 10.1, 95% CI 4.99, 20.5) after controlling for COVID-19 history, 2018 functional or cognitive status, age, gender, marital status, number of chronic conditions, household size, graduation from high school, and self-report of COVID-19 vaccination. There was no association between baseline cognitive status and report of a work-limiting impairment in 2022. Only those reporting CIND in 2018 were more likely to report applying for disability benefits in 2022 (OR 2.85, 95% CI 1.63, 4.97). As with the prior analysis, the effect of 2018 cognitive status on the work-related outcomes was not modified by COVID-19 history (Supplemental Table 3).

## Discussion

In this longitudinal cohort study, situated at the intersection of COVID-19, aging, and disability, we leveraged prospectively collected biannual data from 8,621 older adults in the Health and Retirement Study (HRS) to examine whether a COVID-19 infection in 2020–2021 modified the relationship between 2018 baseline functional or cognitive impairment and subsequent health services use, ability to work, or disability benefits needs. We found no significant interaction: COVID-19 infection history did not exacerbate multiple outcomes in these older adults with pre-existing impairments. While our findings may not support our initial hypothesis, our application of a large, nationally representative cohort with pre-pandemic baseline data offers important insights by distinguishing pre-existing limitations from new or exacerbated symptoms. This design allows for a nuanced perspective on how older adults who lived through the pandemic with varying degrees of impairment might have been impacted. It also addresses a gap in the literature left by cross-sectional or convenience sample studies that lack pre-existing functional and cognitive data.

Though our findings did not support our hypothesis, our findings align with prior research indicating that baseline functional and cognitive impairment are major drivers of healthcare utilization and work limitations [20–23]. The absence of an interaction effect may reflect survival bias. The older adults most vulnerable to severe complications of COVID-19 may have succumbed to the infection rather than surviving with prolonged disability, something which we were able to identify in our analysis which compared those who died or were lost to follow-up between 2018 and 2022. Those who died were more likely to have risk factors for poor outcomes from COVID-19 infection. For example, during the first year of the pandemic, 81% of the deaths from COVID-19 were in those ≥ 65 years of age [24] and in 2023, older adults accounted for 90% of the COVID-19 related in-hospital deaths [25]. This survivor bias may mean that those who did survive might represent a healthier subset of older adults who are less likely to experience severe post-infection complications. Nonetheless, this study provides evidence on how such survivor bias may play a role in shaping the post-pandemic landscape among older adults.

Disentangling the long-term effects of COVID-19 infection from the natural aging process in older adults presents a unique challenge. While older adults may experience outcomes influenced by both infection and age-related decline*,*younger patients may exhibit different patterns and severity of symptoms. For example, in a study of 200 post-hospitalization Neuro-PASC (PNP) and 1,100 non-hospitalized Neuro-PASC (NNP) adult patients identified at a neuro COVID-19 clinic between May 2020 and March 2023, investigators found that 10 months after infection, older patients had a lower prevalence and Neuro-PASC symptom burden than those who were younger or middle aged. In addition, those who were younger or middle aged had a higher prevalence of cognitive dysfunction that led to decreased quality of life (QoL) when compared to older adults [26].

While Long Covid may significantly impact the quality of life (QoL) of older adults [27, 28], it may not necessarily lead to increased health services use beyond what would be expected for older adults as they age. We originally hypothesized that persistent post-infection sequelae among older adults with baseline impairments may manifest in more doctor visits. However, our measure of “seeing a doctor in the last two years,” in an older population where most respondents reported in the affirmative, may not have been sufficiently sensitive to detect incremental changes in outpatient care. We did explore whether the number of doctor visits varied by COVID-19 history but there was no significant difference on bivariable analysis (*p*> 0.90). An unexpected finding was that participants with the most functional limitations were actually less likely to report having seen a doctor in the past two years. It is possible that they faced compounded barriers to outpatient care, such as mobility challenges, limited transportation, or fear of virus exposure. These obstacles may deter or prevent them from seeking timely outpatient care, even if they experience persistent post-infection symptoms. Thus, while Long Covid symptoms may not require hospitalization or extensive medical intervention, inadequate outpatient follow-up among the already vulnerable may place them at risk for further decline. Furthermore, the chronic and often non-acute nature of Long Covid symptoms, such as fatigue and cognitive complaints, might not reach a threshold that requires emergency or inpatient care, similar to patterns observed in other post-viral syndromes and chronic fatigue conditions [5, 29].

The lack of significant interaction between COVID-19 infection history and work-related outcomes in our cohort could be attributed to the retirement status of older adults [30–32]. HRS respondents may have been on the verge of retirement regardless of COVID-19 infection history, and as a result, the experience of COVID-19 would not alter their employment status [33] or lead them to label themselves as disabled. In keeping with our findings of a lack of difference in work outcomes based on COVID-19 infection history in our study population, analysis of employment data from the National Bureau of Economic Research conducted between March 2020 and March 2021 revealed that there was no significant difference in the number of applications for Social Security retired worker benefits [34]. Older workers were also less likely to apply for SSI during the time period. This aligns with the broader understanding that the working-age population might exhibit more pronounced disruptions in work capacity and employment status due to COVID-19 infection compared to older adults nearing or already in retirement [26]. Nevertheless, the persistence of high healthcare utilization and work limitations among respondents with pre-existing impairments suggests that these remain important factors for subsequent healthcare and disability related outcomes.

### Limitations

Our study has several limitations. The self-reported nature of the data introduces the potential for recall bias, and we were unable to independently verify exposures such as baseline functional and cognitive impairment, COVID-19 infection history, and health services utilization outcomes. Furthermore, those who survived to participate in subsequent survey waves may represent a healthier cohort than those who did not survive, potentially skewing our results. While this study attempted to ascertain what the long-term impacts of COVID-19 infection may look like in older adults, we were not able to determine whether individuals in this cohort experienced prolonged post-infection symptoms (i.e., long COVID). In addition, we did not control for cognitive impairment and functional impairment together in the models. Future studies should consider these factors and seek to validate findings through medical records or other objective data sources.

## Conclusions

In conclusion, our study indicates that in a cohort of older adults, the relationship between functional and cognitive impairment and health services use, ability to work, or disability benefits application did not vary based on COVID-19 infection in 2020–2021. These findings highlight that Long Covid may need to be assessed differently in older adults, accounting for the natural aging process, and underscore the need for further research into the unique experiences of older adults with Long Covid, as well as targeted interventions that address the specific needs of this population. Understanding the nuances is critical for informing healthcare services, workforce management, and social service planning to better support older adults in the post-pandemic era.

## Supplementary Information

Supplementary Material 1.

## Data Availability

The datasets used and/or analysed during the current study are available from the corresponding author on reasonable request.
