# Supplementary material for: Health service use and work related outcomes in older adults with functional and cognitive impairments during the COVID-19 pandemic
Source: BMC Public Health. 2025 Nov 7;25:3846. doi: 10.1186/s12889-025-25129-2 (PMC12598834; doi:10.1186/s12889-025-25129-2)
Supplement: Supplementary file 1 — Supplementary Material 1. [file 12889_2025_25129_MOESM1_ESM.docx]

**Supplemental Tables**

**Supplemental Table 1.** Baseline characteristics of those who participated in 2022 survey wave versus those who died or were lost to follow-up

**Supplemental Table 2.** Healthcare utilization outcome models including functional limitation and cognitive limitation, and interaction with COVID-19 status

**Supplemental Table 3.** Work related outcome models including functional limitation and cognitive limitation, and interaction with COVID-19 status

**Supplemental Table 1.** Baseline characteristics of those who participated in 2022 survey wave versus those who died or were lost to follow-up

|  | **Status in 2022** | | |  |
| --- | --- | --- | --- | --- |
| **Characteristic** | **Overall**  N = 9714  n (%)^1^ | **Died**  N = 1093  n (%)^1^ | **Alive**  N = 8621  n (%)^1^ | **p-value**^2^ |
| Age, years, median (IQR) | 65 (59, 72) | 65 (58, 74) | 65 (59, 71) | 0.041 |
| Female | 5,802 (55%) | 641 (57%) | 5,161 (55%) | 0.23 |
| Race |  |  |  | 0.26 |
| White/Caucasian | 6,866 (83%) | 788 (82%) | 6,078 (83%) |  |
| Black/African American | 1,873 (8.7%) | 183 (7.9%) | 1,690 (8.8%) |  |
| Other | 946 (8.1%) | 121 (10.0%) | 825 (7.8%) |  |
| Hispanic | 1,299 (7.8%) | 141 (8.0%) | 1,158 (7.7%) | 0.83 |
| Completed at least high school | 8,338 (91%) | 914 (87%) | 7,424 (91%) | 0.011 |
| Married | 6,249 (69%) | 670 (64%) | 5,579 (70%) | 0.004 |
| Hypertension | 5,889 (55%) | 685 (57%) | 5,204 (55%) | 0.39 |
| Diabetes | 2,569 (23%) | 297 (24%) | 2,272 (23%) | 0.53 |
| Cancer | 1,460 (14%) | 203 (17%) | 1,257 (14%) | 0.013 |
| Lung disease | 972 (9.1%) | 147 (13%) | 825 (8.6%) | <0.001 |
| Heart problems | 2,234 (22%) | 301 (25%) | 1,933 (21%) | 0.021 |
| Stroke | 693 (6.0%) | 119 (9.8%) | 574 (5.5%) | <0.001 |
| Emotional/psychiatric problems | 2,030 (21%) | 242 (22%) | 1,788 (21%) | 0.76 |
| Arthritis | 5,841 (58%) | 676 (57%) | 5,165 (58%) | 0.92 |
| Total # of comorbidities, mean (SD) | 2.07 (1.46) | 2.25 (1.59) | 2.05 (1.45) | 0.039 |
| Smoking |  |  |  | 0.001 |
| Never | 4,572 (48%) | 460 (42%) | 4,112 (49%) |  |
| Former | 4,041 (42%) | 486 (44%) | 3,555 (42%) |  |
| Current | 1,070 (10%) | 146 (14%) | 924 (9.9%) |  |
| ^1^All counts unweighted and percentages are weighted | | | | |
| ^2^Design-based KruskalWallis test; Pearson's X^2: Rao & Scott adjustment | | | | |

**Supplemental Table 2.** Healthcare utilization outcome models including functional limitation and cognitive limitation, and interaction with COVID-19 status

**Functional Limitation**

***Ever stayed in a nursing home in last 2 years: interaction***

| **Characteristic** | **OR** | **95% CI** | **p-value** |
| --- | --- | --- | --- |
| **Positive COVID History** | 1.33 | 0.42, 4.21 | 0.628 |
| **Functional Limitations, 2018** |  |  |  |
| Less | — | — |  |
| Moderate | 2.96 | 1.54, 5.69 | 0.001 |
| Most | 7.28 | 3.60, 14.7 | <0.001 |
| **Age, y.** | 1.08 | 1.06, 1.11 | <0.001 |
| **Female gender** | 0.76 | 0.53, 1.11 | 0.153 |
| **Marital Status, 2018** |  |  |  |
| Married/partnered | — | — |  |
| Not married/not partnered | 2.00 | 1.07, 3.77 | 0.032 |
| **No. chronic conditions** | 1.21 | 1.08, 1.37 | 0.002 |
| **Household size** | 0.94 | 0.76, 1.16 | 0.572 |
| **Graduated high school** | 1.00 | 0.61, 1.66 | 0.989 |
| **Had a COVID-19 vaccine** | 0.83 | 0.45, 1.53 | 0.539 |
| **Positive COVID History * Functional Limitations, 2018** |  |  |  |
| Positive COVID History * Moderate | 1.36 | 0.36, 5.12 | 0.640 |
| Positive COVID History * Most | 1.47 | 0.36, 5.93 | 0.585 |
| Abbreviations: CI = Confidence Interval, OR = Odds Ratio  P-value of Rao-Scott LRT of interaction is 0.820. | | | |

***Ever stayed in a hospital in the last 2 yrs: interaction***

| **Characteristic** | **OR** | **95% CI** | **p-value** |
| --- | --- | --- | --- |
| **Positive COVID History** | 1.34 | 1.04, 1.74 | 0.024 |
| **Functional Limitations, 2018** |  |  |  |
| Less | — | — |  |
| Moderate | 1.52 | 1.25, 1.84 | <0.001 |
| Most | 2.13 | 1.59, 2.84 | <0.001 |
| **Age, y.** | 1.03 | 1.02, 1.04 | <0.001 |
| **Female gender** | 0.72 | 0.63, 0.83 | <0.001 |
| **Marital Status, 2018** |  |  |  |
| Married/partnered | — | — |  |
| Not married/not partnered | 1.15 | 0.96, 1.37 | 0.135 |
| **No. chronic conditions** | 1.24 | 1.19, 1.29 | <0.001 |
| **Household size** | 1.00 | 0.92, 1.09 | 0.974 |
| **Graduated high school** | 1.26 | 1.02, 1.56 | 0.036 |
| **Had a COVID-19 vaccine** | 1.07 | 0.82, 1.39 | 0.624 |
| **Positive COVID History * Functional Limitations, 2018** |  |  |  |
| Positive COVID History * Moderate | 1.16 | 0.80, 1.69 | 0.429 |
| Positive COVID History * Most | 0.77 | 0.49, 1.22 | 0.263 |
| Abbreviations: CI = Confidence Interval, OR = Odds Ratio  P-value of Rao-Scott LRT of interaction is 0.247. | | | |

***Seen a doctor at least once in the last 2 yrs: interaction***

| **Characteristic** | **OR** | **95% CI** | **p-value** |
| --- | --- | --- | --- |
| **Positive COVID History** | 1.15 | 0.77, 1.73 | 0.491 |
| **Functional Limitations, 2018** |  |  |  |
| Less | — | — |  |
| Moderate | 1.17 | 0.88, 1.56 | 0.271 |
| Most | 1.52 | 0.97, 2.39 | 0.069 |
| **Age, y.** | 1.01 | 1.00, 1.02 | 0.216 |
| **Female gender** | 1.48 | 1.19, 1.84 | <0.001 |
| **Marital Status, 2018** |  |  |  |
| Married/partnered | — | — |  |
| Not married/not partnered | 0.45 | 0.35, 0.59 | <0.001 |
| **No. chronic conditions** | 1.32 | 1.18, 1.48 | <0.001 |
| **Household size** | 0.84 | 0.76, 0.92 | <0.001 |
| **Graduated high school** | 3.77 | 2.87, 4.96 | <0.001 |
| **Had a COVID-19 vaccine** | 1.68 | 1.23, 2.31 | 0.002 |
| **Positive COVID History * Functional Limitations, 2018** |  |  |  |
| Positive COVID History * Moderate | 0.90 | 0.45, 1.79 | 0.757 |
| Positive COVID History * Most | 0.43 | 0.20, 0.94 | 0.036 |
| Abbreviations: CI = Confidence Interval, OR = Odds Ratio  P-value of Rao-Scott LRT of interaction is 0.150. | | | |

**Cognitive Limitation**

***Ever stayed in a hospital in the last 2 yrs: interaction***

| **Characteristic** | **OR** | **95% CI** | **p-value** |
| --- | --- | --- | --- |
| **Positive COVID History** | 1.39 | 1.16, 1.66 | <0.001 |
| **Cognitive Function, 2018** |  |  |  |
| Normal | — | — |  |
| CIND | 1.26 | 0.95, 1.66 | 0.108 |
| Dementia | 0.99 | 0.55, 1.78 | 0.960 |
| **Age, y.** | 1.03 | 1.02, 1.04 | <0.001 |
| **Female gender** | 0.77 | 0.67, 0.88 | <0.001 |
| **Marital Status, 2018** |  |  |  |
| Married/partnered | — | — |  |
| Not married/not partnered | 1.22 | 1.02, 1.45 | 0.029 |
| **No. chronic conditions** | 1.33 | 1.27, 1.39 | <0.001 |
| **Household size** | 1.01 | 0.93, 1.09 | 0.897 |
| **Graduated high school** | 1.19 | 0.95, 1.49 | 0.120 |
| **Had a COVID-19 vaccine** | 1.04 | 0.81, 1.34 | 0.744 |
| **Positive COVID History * Cognitive Function, 2018** |  |  |  |
| Positive COVID History * CIND | 0.67 | 0.39, 1.15 | 0.143 |
| Positive COVID History * Dementia | 1.81 | 0.54, 6.08 | 0.334 |
| Abbreviations: CI = Confidence Interval, OR = Odds Ratio  P-value of Rao-Scott LRT of interaction is 0.222. | | | |

***Seen a doctor at least once in the last 2 yrs: interaction***

| **Characteristic** | **OR** | **95% CI** | **p-value** |
| --- | --- | --- | --- |
| **Positive COVID History** | 1.02 | 0.75, 1.40 | 0.888 |
| **Cognitive Function, 2018** |  |  |  |
| Normal | — | — |  |
| CIND | 0.58 | 0.42, 0.81 | 0.002 |
| Dementia | 0.46 | 0.21, 1.01 | 0.053 |
| **Age, y.** | 1.01 | 1.00, 1.02 | 0.102 |
| **Female gender** | 1.49 | 1.19, 1.85 | <0.001 |
| **Marital Status, 2018** |  |  |  |
| Married/partnered | — | — |  |
| Not married/not partnered | 0.47 | 0.36, 0.62 | <0.001 |
| **No. chronic conditions** | 1.37 | 1.25, 1.49 | <0.001 |
| **Household size** | 0.84 | 0.77, 0.93 | <0.001 |
| **Graduated high school** | 3.22 | 2.38, 4.36 | <0.001 |
| **Had a COVID-19 vaccine** | 1.65 | 1.21, 2.26 | 0.002 |
| **Positive COVID History * Cognitive Function, 2018** |  |  |  |
| Positive COVID History * CIND | 1.00 | 0.41, 2.44 | 0.991 |
| Positive COVID History * Dementia | 1.05 | 0.27, 4.07 | 0.944 |
| Abbreviations: CI = Confidence Interval, OR = Odds Ratio  P-value of Rao-Scott LRT of interaction is 0.998. | | | |

**Supplemental Table 3.** Work related outcome models including functional limitation and cognitive limitation, and interaction with COVID-19 status

**Functional Limitation**

***Unable to work, 2022: interaction***

| **Characteristic** | **OR** | **95% CI** | **p-value** |
| --- | --- | --- | --- |
| **Positive COVID History** | 0.65 | 0.32, 1.32 | 0.231 |
| **Functional Limitations, 2018** |  |  |  |
| Less | — | — |  |
| Moderate | 4.78 | 2.75, 8.30 | <0.001 |
| Most | 30.0 | 16.6, 54.1 | <0.001 |
| **Age, y.** | 0.90 | 0.88, 0.92 | <0.001 |
| **Female gender** | 0.57 | 0.43, 0.75 | <0.001 |
| **Marital Status, 2018** |  |  |  |
| Married/partnered | — | — |  |
| Not married/not partnered | 2.17 | 1.66, 2.83 | <0.001 |
| **Number of chronic conditions** | 1.54 | 1.37, 1.72 | <0.001 |
| **Household size** | 0.98 | 0.86, 1.12 | 0.757 |
| **Graduated high school** | 0.43 | 0.31, 0.61 | <0.001 |
| **Had a COVID-19 vaccine** | 0.78 | 0.46, 1.33 | 0.357 |
| **Positive COVID History * Functional Limitations, 2018** |  |  |  |
| Positive COVID History * Moderate | 1.31 | 0.49, 3.50 | 0.582 |
| Positive COVID History * Most | 1.46 | 0.61, 3.53 | 0.389 |
| Abbreviations: CI = Confidence Interval, OR = Odds Ratio  P-value of Rao-Scott LRT of interaction is 0.715. | | | |

***Work-limiting impairment, 2022: interaction***

| **Characteristic** | **OR** | **95% CI** | **p-value** |
| --- | --- | --- | --- |
| **Positive COVID History** | 0.96 | 0.65, 1.43 | 0.851 |
| **Functional Limitations, 2018** |  |  |  |
| Less | — | — |  |
| Moderate | 4.87 | 3.59, 6.61 | <0.001 |
| Most | 10.8 | 6.45, 17.9 | <0.001 |
| **Age, y.** | 1.00 | 0.98, 1.02 | 0.714 |
| **Female gender** | 1.01 | 0.75, 1.34 | 0.967 |
| **Marital Status, 2018** |  |  |  |
| Married/partnered | — | — |  |
| Not married/not partnered | 1.26 | 0.95, 1.68 | 0.114 |
| **Number of chronic conditions** | 1.41 | 1.28, 1.56 | <0.001 |
| **Household size** | 0.93 | 0.83, 1.04 | 0.218 |
| **Graduated high school** | 0.89 | 0.52, 1.50 | 0.655 |
| **Had a COVID-19 vaccine** | 0.66 | 0.43, 1.0 | 0.047 |
| **Positive COVID History * Functional Limitations, 2018** |  |  |  |
| Positive COVID History * Moderate | 1.14 | 0.63, 2.07 | 0.666 |
| Positive COVID History * Most | 1.22 | 0.49, 3.01 | 0.667 |
| Abbreviations: CI = Confidence Interval, OR = Odds Ratio  P-value of Rao-Scott LRT of interaction is 0.863. | | | |

***Applied for SSI, 2022: interaction***

| **Characteristic** | **OR** | **95% CI** | **p-value** |
| --- | --- | --- | --- |
| **Positive COVID History** | 0.56 | 0.19, 1.68 | 0.299 |
| **Functional Limitations, 2018** |  |  |  |
| Less | — | — |  |
| Moderate | 1.24 | 0.56, 2.78 | 0.593 |
| Most | 2.39 | 0.97, 5.86 | 0.057 |
| **Age, y.** | 0.93 | 0.89, 0.97 | 0.001 |
| **Female gender** | 0.59 | 0.36, 0.97 | 0.039 |
| **Marital Status, 2018** |  |  |  |
| Married/partnered | — | — |  |
| Not married/not partnered | 2.69 | 1.58, 4.56 | <0.001 |
| **Number of chronic conditions** | 1.32 | 1.08, 1.60 | 0.006 |
| **Household size** | 1.13 | 0.97, 1.33 | 0.112 |
| **Graduated high school** | 0.39 | 0.21, 0.72 | 0.003 |
| **Had a COVID-19 vaccine** | 0.42 | 0.23, 0.77 | 0.005 |
| **Positive COVID History * Functional Limitations, 2018** |  |  |  |
| Positive COVID History * Moderate | 2.27 | 0.57, 8.98 | 0.239 |
| Positive COVID History * Most | 1.70 | 0.43, 6.80 | 0.444 |
| Abbreviations: CI = Confidence Interval, OR = Odds Ratio  P-value of Rao-Scott LRT of interaction is 0.472. | | | |

**Cognitive Limitation**

***Unable to work, 2022: interaction***

| **Characteristic** | **OR** | **95% CI** | **p-value** |
| --- | --- | --- | --- |
| **Positive COVID History** | 0.84 | 0.62, 1.15 | 0.272 |
| **Cognitive Status, 2018** |  |  |  |
| Normal | — | — |  |
| CIND | 2.32 | 1.55, 3.48 | <0.001 |
| Dementia | 8.24 | 3.93, 17.3 | <0.001 |
| **Age, y.** | 0.90 | 0.88, 0.92 | <0.001 |
| **Female gender** | 0.78 | 0.60, 1.02 | 0.065 |
| **Marital Status, 2018** |  |  |  |
| Married/partnered | — | — |  |
| Not married/not partnered | 2.45 | 1.89, 3.19 | <0.001 |
| **Number of chronic conditions** | 2.18 | 2.01, 2.38 | <0.001 |
| **Household size** | 0.97 | 0.86, 1.09 | 0.588 |
| **Graduated high school** | 0.45 | 0.33, 0.62 | <0.001 |
| **Had a COVID-19 vaccine** | 0.78 | 0.49, 1.25 | 0.297 |
| **Positive COVID History * Cognitive Status, 2018** |  |  |  |
| Positive COVID History * CIND | 1.03 | 0.46, 2.30 | 0.939 |
| Positive COVID History * Dementia | 2.07 | 0.48, 8.95 | 0.324 |
| Abbreviations: CI = Confidence Interval, OR = Odds Ratio  P-value of Rao-Scott LRT of interaction is 0.600. | | | |

***Work-limiting impairment, 2022: interaction***

| **Characteristic** | **OR** | **95% CI** | **p-value** |
| --- | --- | --- | --- |
| **Positive COVID History** | 0.94 | 0.73, 1.21 | 0.636 |
| **Cognitive Status, 2018** |  |  |  |
| Normal | — | — |  |
| CIND | 0.96 | 0.59, 1.55 | 0.853 |
| Dementia | 1.01 | 0.36, 2.89 | 0.978 |
| **Age, y.** | 1.00 | 0.98, 1.01 | 0.694 |
| **Female gender** | 1.18 | 0.90, 1.54 | 0.230 |
| **Marital Status, 2018** |  |  |  |
| Married/partnered | — | — |  |
| Not married/not partnered | 1.48 | 1.14, 1.91 | 0.003 |
| **Number of chronic conditions** | 1.82 | 1.67, 1.98 | <0.001 |
| **Household size** | 0.96 | 0.88, 1.06 | 0.420 |
| **Graduated high school** | 0.80 | 0.47, 1.36 | 0.402 |
| **Had a COVID-19 vaccine** | 0.67 | 0.45, 0.99 | 0.043 |
| **Positive COVID History * Cognitive Status, 2018** |  |  |  |
| Positive COVID History * CIND | 2.46 | 0.92, 6.56 | 0.072 |
| Positive COVID History * Dementia | 2.28 | 0.39, 13.5 | 0.359 |
| Abbreviations: CI = Confidence Interval, OR = Odds Ratio  P-value of Rao-Scott LRT of interaction is 0.098. | | | |

***Applied for SSI, 2022: interaction***

| **Characteristic** | **OR** | **95% CI** | **p-value** |
| --- | --- | --- | --- |
| **Positive COVID History** | 1.04 | 0.50, 2.14 | 0.918 |
| **Cognitive Status, 2018** |  |  |  |
| Normal | — | — |  |
| CIND | 3.27 | 1.75, 6.09 | <0.001 |
| Dementia | 1.75 | 0.59, 5.23 | 0.309 |
| **Age, y.** | 0.93 | 0.89, 0.97 | <0.001 |
| **Female gender** | 0.63 | 0.39, 1.03 | 0.065 |
| **Marital Status, 2018** |  |  |  |
| Married/partnered | — | — |  |
| Not married/not partnered | 2.78 | 1.68, 4.60 | <0.001 |
| **Number of chronic conditions** | 1.47 | 1.24, 1.74 | <0.001 |
| **Household size** | 1.12 | 0.97, 1.28 | 0.121 |
| **Graduated high school** | 0.42 | 0.23, 0.77 | 0.006 |
| **Had a COVID-19 vaccine** | 0.41 | 0.23, 0.75 | 0.005 |
| **Positive COVID History * Cognitive Status, 2018** |  |  |  |
| Positive COVID History * CIND | 0.52 | 0.15, 1.76 | 0.291 |
| Positive COVID History * Dementia | 0.31 | 0.03, 3.64 | 0.343 |
| Abbreviations: CI = Confidence Interval, OR = Odds Ratio | | | |

P-value of Rao-Scott LRT of interaction is 0.346.
